# Handgrip Strength and Trajectories of Preclinical Obesity Progression: A Multistate Model Analysis Using the UK Biobank

**DOI:** 10.1210/clinem/dgaf521

**Published:** 2026-02-20

**Authors:** Manrong Xu, Menghan Li, Yawen Zhang, Lianxi Li, Yun Shen, Gang Hu

**Affiliations:** 1 Department of Endocrinology and Metabolism, Shanghai Sixth People’s Hospital Affiliated to Shanghai Jiao Tong University School of Medicine, Shanghai Clinical Center for Diabetes, Shanghai Diabetes Institute, Shanghai Key Laboratory of Diabetes Mellitus, Shanghai Key Clinical Center for Metabolic Disease, Shanghai 200233, China; 2 Chronic Disease Epidemiology Laboratory, Pennington Biomedical Research Center, Baton Rouge, LA 70808, USA

**Keywords:** preclinical obesity, grip strength, obesity-induced dysfunctions, all-cause mortality, multistate model, UK biobank

## Abstract

**Purpose:**

Grip strength has been increasingly recognized as a predictor of chronic disease risk and mortality. The aim of our study was to investigate the association of grip strength and the trajectories of preclinical obesity progression.

**Methods:**

Data were collected from 93 275 participants in the UK Biobank. Preclinical obesity was diagnosed based on an excess of anthropometric parameters, defined as elevated body mass index combined with at least 1 abnormal measure among waist circumference, waist-to-hip ratio, waist-to-height ratio, or percentage body fat, in the absence of obesity-induced dysfunctions. Three models captured different trajectories from baseline to dysfunctions and death, with or without intermediate progression. A multistate model was used to investigate the association between grip strength and the preclinical obesity progression and multiple-cause mortality risk. Sensitivity analyses were performed using free muscle volume, total lean mass, and muscle-to-weight ratio as exposures.

**Results:**

Among 8163 death events over a mean follow-up of 13.4 years, each SD increase in grip strength was associated with a significantly reduced risk of preclinical obesity progression at each stage, with the strongest inverse association observed in baseline to first dysfunction [fully adjusted hazard ratio (HR): 0.86, 95% confidence interval (CI): 0.85–0.88]. Compared to the lowest tertile, the highest grip strength significantly showed protective effects across all trajectory models, with double dysfunctions to all-cause death yielding the most pronounced associations (fully adjusted HR: 0.77, 95% CI: 0.70–0.84). Further subgroup and sensitivity analysis showed consistent results.

**Conclusion:**

Increased grip strength was significantly associated with a decreased risk of obesity-induced dysfunctions progression and multiple-cause mortality. These findings underscore the importance of improving muscle mass and strength in preclinical obesity.

Currently, body mass index (BMI) remains the primary metric for diagnosing obesity. However, BMI does not differentiate between fat and lean mass or capture variations in body composition and fat distribution (1). Consequently, BMI-based obesity definitions may inaccurately estimate actual adiposity, particularly excess fat accumulation, across diverse age groups, sexes, and ethnic populations (2, 3). For instance, a study using dual-energy X-ray absorptiometry (DXA) showed that obesity prevalence determined by total body fat percentage was markedly higher among young and middle-aged adults compared to BMI-based definitions (2). Conversely, elevated BMI values among athletes may primarily reflect increased muscle mass rather than excess adiposity.

Recent evidence underscores muscle strength and function as potentially better indicators of health risks associated with obesity (4, 5). Muscle weakness has been independently associated with central obesity and increased risks of physical disability and chronic disease outcomes. Declines in muscle mass and strength have also been linked with higher risks of obesity-related complications, including increased cardiovascular and all-cause mortality (6, 7). For example, low grip strength has been identified as an independent predictor of mortality risk among individuals over age 50, regardless of BMI-defined obesity (8). Interestingly, among individuals over age 70, overweight and obesity by BMI criteria were associated with protective effects against mortality (8), highlighting the limitations of anthropometric-based obesity measures in accurately predicting clinical outcomes.

In January 2025, a consensus statement published in *The Lancet Diabetes & Endocrinology* formally recognized obesity as a clinical disease, emphasizing the direct detrimental effects of excessive adiposity on organ function, systemic health, and limitations in activities of daily living (ADLs) (9). The concept of “preclinical obesity” emerged, defined as having excess anthropometric measures without overt obesity-related dysfunctions (9). The Lancet commissioners advocated for a “real obesity” status, emphasizing its significance in public health. Following the publication of the Lancet Commission’s report, we assessed the prevalence of clinical obesity in the US population and identified a higher risk of incident cancer associated with obesity defined by these new criteria (10, 11). However, the potential role of muscle strength in influencing the transition from preclinical obesity to overt dysfunction and subsequent mortality remains poorly understood.

To date, few studies have specifically investigated how muscle strength may influence the progression trajectories from preclinical obesity to clinical obesity and associated outcomes. Therefore, the present study aimed to investigate whether grip strength was associated with the progression from preclinical obesity to clinical obesity and whether grip strength could reduce the risk of mortality among individuals progressing through obesity-related dysfunction states.

## Methods

### Research Design and Methods

**Figure 1.**
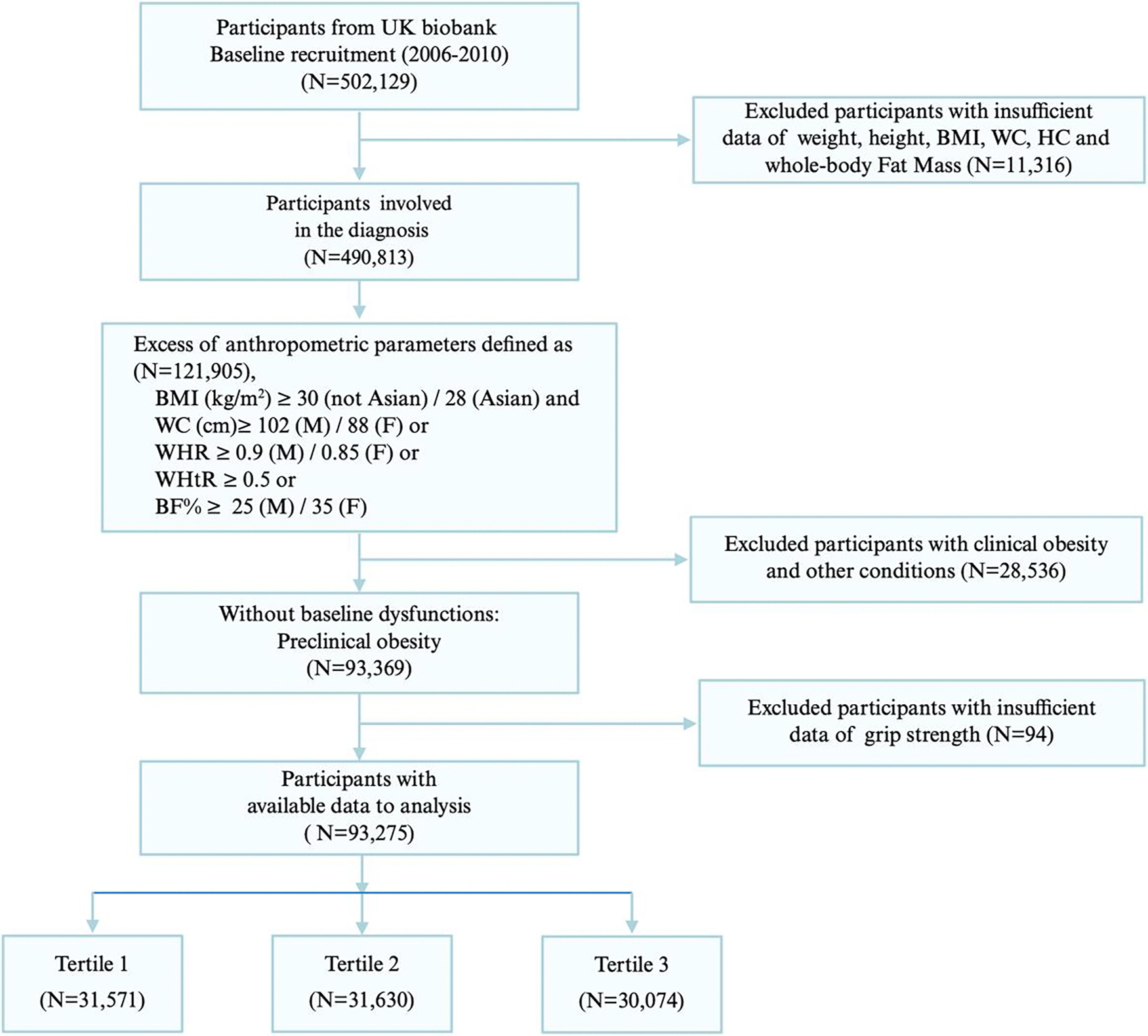
Flow chart of the study participants’ selection process. T1, T2, and T3 represent sex-specific tertiles of grip strength: men: T1 (≤36.5 kg), T2 (36.5–44.0 kg), T3 (>44.0 kg); women: T1 (≤20.5 kg), T2 (20.5–26.0 kg), T3 (>26.0 kg).

This prospective cohort study was based on data from the UK Biobank, a nationally representative and continuously updated biomedical database in the United Kingdom. The cohort comprises over 500 000 participants who were recruited between 2006 and 2010 from 3 assessment centers located in England, Wales, and Scotland. The UK Biobank project received ethical approval from the National Health Service’s National Research Ethics Service. The current study protocol was approved by the UK Biobank data access committee. As shown in Fig. 1, a total of 502 129 participants were initially screened from the full UK Biobank dataset. Individuals lacking data on BMI, waist circumference (WC), hip circumference, and whole-body fat mass measured by bioelectrical impedance (n = 11 316) were excluded from the analysis. The diagnostic criteria for an excess of anthropometric parameters are as follows. First, BMI should meet the criteria: for non-Asian populations, BMI (kg/cm^2^) ≥ 30 or for Asian populations, BMI ≥ 28 (12), and 1 of the following conditions must be identified: (1) for men, WC (cm) ≥ 102 or for women, WC ≥ 88 (13); (2) for men, waist-to-hip ratio ≥ 0.9 or for women, waist-to-hip ≥ 0.85 (13); (3) waist-to-height ratio ≥ 0.5 (14); (4) percentage body fat, calculated as (whole-body fat mass/body weight) × 100%: ≥ 25 for men or ≥35 for women (15). The dysfunctions due to obesity (obesity-induced dysfunctions) based on the recent consensus (9) were identified by International Classification of Diseases 10th Revision (ICD-10) codes, including the limitation of ADL (listed in Table 1).

If the patient with an excess of anthropometric parameters had been diagnosed with any of the 18 dysfunctions, they were diagnosed with clinical obesity. Preclinical obesity was defined by individuals with an excess of anthropometric parameters who had not developed any dysfunctions due to obesity at baseline. Participants with clinical obesity and other conditions were excluded (n = 28 536). Ultimately, after excluding participants with insufficient data of grip strength (n = 94) in preclinical obesity, a total of 93 275 participants were retained for the final analysis (see flowchart in Fig. 1).

### Exposure and Other Covariates

The grip strength of both the left and right hands was measured separately using a Jamar J00105 hydraulic hand dynamometer, and the average value of both hands was used for analysis (16). The study population was then categorized into 3 groups according to sex-specific tertiles of grip strength (men: ≤ 36.5 kg, 36.5–44.0 kg, and >44.0 kg; women: ≤ 20.5 kg, 20.5–26.0 kg, and >26.0 kg).

Total thigh fat-free muscle volume was measured by magnetic resonance imaging (MRI), and total lean mass was assessed by DXA. MRI was conducted using a Siemens MAGNETOM Aera 1.5T scanner and DXA using the GE-Lunar iDXA scanner (17, 18). MRI and DXA data were available for a subset of participants in our study (n = 6023 for MRI and n = 6380 for DXA). The muscle-to-weight ratio (MWR) was defined as the ratio of total thigh fat-free muscle volume to body weight, while the lean-to-weight ratio (LWR) was defined as the ratio of total lean mass to body weight.

In the UK Biobank cohort, lifestyle and behavioral characteristics were assessed via a touchscreen questionnaire. Participants reported their alcohol consumption and smoking status, which were categorized as “never,” “previous,” or “current”; both variables also included a “prefer not to answer” option. Baseline demographic and clinical data included age, sex, ethnicity, education level, and employment status. Family history of diabetes was defined as having either biological parent diagnosed with diabetes. Regular physical activity was defined as engaging in at least 150 minutes per week of moderate-intensity activity, 75 minutes per week of vigorous-intensity activity, or an equivalent combination of both (19). Sleep duration was dichotomized into ≥6 hours and <6 hours. Healthy diet was defined as achieving 5 or more adequate intakes, where adequate intakes were defined as increased consumption of fruits, vegetables, whole grains, fish, dairy, and vegetable oils, as well as reduced consumption of refined grains, processed/unprocessed meats, and sugar-sweetened beverages (20). Self-reported regular use of medications was recorded for several drug categories, including lipid-lowering agents, antihypertensive medications, and glucose-lowering therapies. Vitamin supplement was classified based on the questionnaire response to “Do you regularly take any of the following” as either having supplements or not. Additionally, baseline physical examination parameters included systolic blood pressure (SBP) and diastolic blood pressure (DBP). The baseline serum or plasma biomarkers were obtained through biochemical testing, including hemoglobin A1c (HbA1c), total cholesterol (TC), low-density lipoprotein cholesterol (LDL), high-density lipoprotein cholesterol (HDL), triglycerides (TG), and C-reactive protein (CRP). Estimated glomerular filtration rate (eGFR) was calculated based on serum creatinine using the sex-specific CKD-EPI equation. Data were used in the present analysis as covariates.

### Assessment of Outcomes

**Figure 2.**
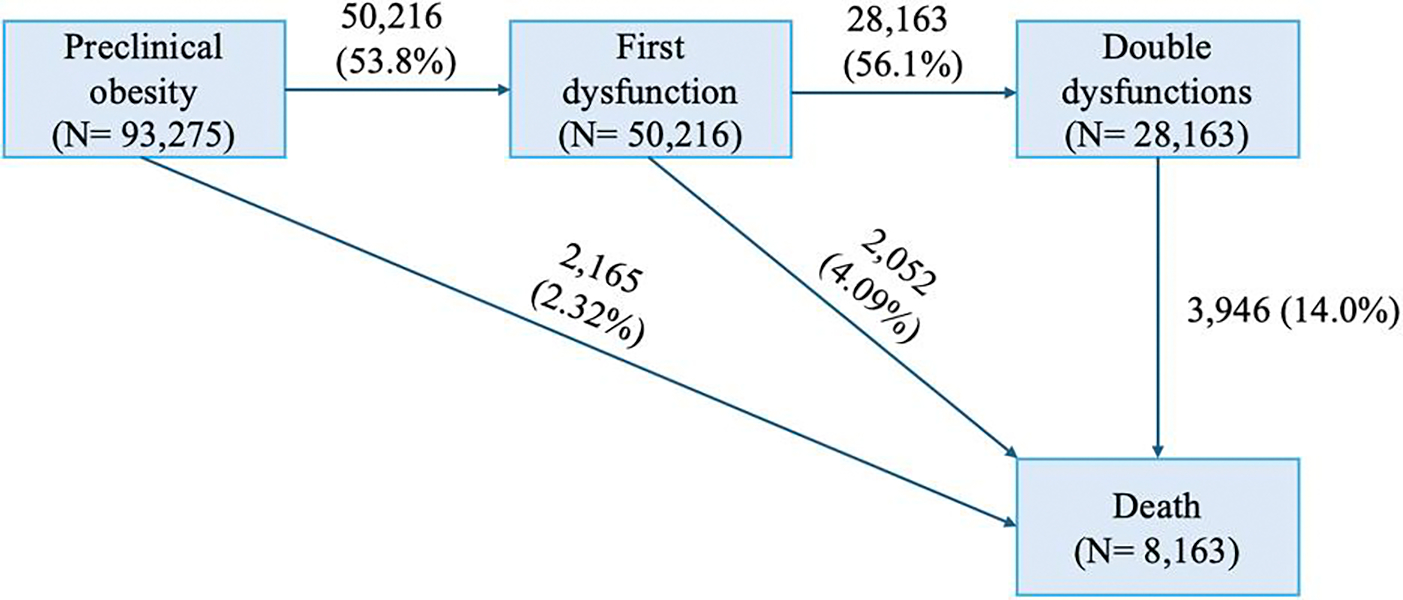
Three transition stages based on the progression trajectory of obesity-induced dysfunctions progression: Model 1: baseline to first obesity-induced dysfunction, first to double obesity-induced dysfunction, and double to all-cause death. Model 2: baseline to first obesity-induced dysfunction, first obesity-induced dysfunction to all-cause death. Model 3: baseline to all-cause death.

The primary outcome of interest was the incidence of new-onset dysfunctions due to obesity and all-cause mortality. Three models were constructed to show the different trajectory of the progression of preclinical obesity (Fig. 2). Model 1 included 3 transitions: from baseline to first obesity-induced dysfunction (n = 50 216; 53.8%), from first to double obesity-induced dysfunction (n = 28 163; 56.1%), and from double obesity-induced dysfunction to all-cause death (n = 3946; 14.0%). Model 2 included 2 transitions: from baseline to first obesity-induced dysfunction (n = 50 216; 53.8%) and from first obesity-induced dysfunction (with no further progression) to all-cause death (n = 2052; 4.09%). Model 3 included a single transition: from baseline, without developing any obesity-induced dysfunction, to all-cause death (n = 2165; 2.32%). According to ICD-10 codes, deaths from cardiovascular disease (CVD) and cancer were defined by I00–I99 and C00–C97 (excluding C44), respectively. The follow-up period for all participants commenced on the date they provided consent to participate in the UK Biobank study and continued until the earliest of the following events: the occurrence of an outcome event, loss to follow-up, or the conclusion of the study follow-up period (21).

### Statistical Analysis

For continuous variables with missing data ≤5%, simple mean imputation was applied. When the missing data rate exceeded 5%, multiple imputation using chained equations was conducted with the predictive mean matching method via the “mice” package in R. Missing data for categorical variables were handled by introducing a separate “missing” category. Normality of continuous variables was assessed using the Shapiro–Wilk test. Histograms and Q–Q plots were also used for visual inspection. Normally distributed data were presented as mean ± SD, while nonnormally distributed data were expressed as median (interquartile range). Categorical variables were reported as frequencies and percentages. One-way ANOVA was used to compare normally distributed variables across clusters, while the Kruskal–Wallis test was employed for nonnormally distributed variables, and the chi-square test was used to compare categorical variables.

In the present study, a multistate model was conducted to investigate the role of grip strength in the temporal progression from preclinical obesity to the development of single and multiple obesity-related dysfunctions, as well as all-cause, CVD-specific, and cancer-specific mortality. For participants who transitioned through multiple stages on the same date, the entry date for the preceding stage was set 0.5 days earlier than that of the subsequent stage, following established methodological conventions (22). Thus, each dysfunction onset was considered as 1 transition. Double dysfunctions therefore may include 2 or more obesity-induced dysfunctions, representing a transition into a multimorbidity state. Based on that, transition-specific multivariable Cox regression was employed to estimate hazard ratios (HRs) to analyze the association between grip strength (entered as a continuous variable per SD increase) and grip strength tertiles and 3 models of different trajectories of preclinical obesity progression. To control for potential confounders, 2 levels of adjustment were applied. In the simple adjustment model, we adjusted for age, sex, and race. In multiple adjustment model, we further adjusted for TG, TC, LDL, HDL, HbA1c, SBP, DBP, BMI, WC, eGFR, and CRP; family history of diabetes; education qualification; employment status; smoking status; alcohol drinking status; healthy diet; regular physical activity; sleep condition; vitamin supplement; and use of antihypertensive drugs, lipid-lowering drugs, and glucose-lowering drugs.

Additionally, subgroup analyses of HRs were conducted for the three disease transitions in model 1, stratified by categorical variables related to lifestyle, family history, diet, physical activity, and socioeconomic status, using per SD increase in grip strength as the exposure. Sensitivity analyses were performed to assess the robustness of the findings. Participants with complete data on either MWR or LWR were analyzed separately. Associations between per SD increase in MWR and LWR and the risk of obesity-induced dysfunctions progression and all-cause mortality were examined.

A *P*-value < .05 was considered statistically significant. All statistical analyses were conducted using R statistical software (version 4.2.2).

## Results

### Baseline Characteristics of the Participants

Table 2 presents participants’ baseline characteristics stratified by sex-specific tertiles of grip strength. With increasing grip strength tertiles, there were significant decreased trends in age, WC, SBP, HbA1c, TG, and CRP, as well as in the proportions of individuals of Black race; those without a college or higher degree; those not currently employed; current or past smokers; individuals adhering to a healthy diet; vitamins supplement; and the use of lipid-lowering, antihypertensive, and glucose-lowering medications. Conversely, DBP, TC, LDL, eGFR, and the proportions of individuals of Asian race, individuals with family history of diabetes, and current drinkers increased with higher grip strength tertiles. The highest sleep duration level (7.05 ± 1.34), HDL level (1.33 ± 0.34) and the largest proportion of individuals of White race [28 080 (88.8%)] were observed in the second tertile. No significant differences were observed across tertiles for sex (*P* = 0.147).

### Association of Grip Strength With Preclinical Progression

During a mean follow-up of 13.4 years, a total of 8163 deaths were recorded. Table 3 showed that with each SD increase in grip strength, the risk of progression across each stage of preclinical obesity was significantly reduced in models 1, 2, and 3. In model 1, the fully adjusted HRs for the 3 progressions were 0.86 [95% confidence interval (CI): 0.85–0.88], 0.92 (95% CI: 0.90–0.94), and 0.87 (95% CI: 0.83–0.91), respectively. In model 2, the fully adjusted HRs for the 2 progressions were 0.86 (95% CI: 0.85–0.88) and 0.93 (95% CI: 0.87–0.99), respectively. Additionally, even in the absence of any obesity-induced dysfunctions, the inverse association between standardized grip strength and the risk of transitioning from baseline to all-cause death remained significant (model 3), with a fully adjusted HR of 0.91 (95% CI: 0.85–0.97).

In addition, grip strength was inversely associated with the risk of preclinical obesity progression in both biological sexes (Table 4). Among men, the most prominent protective association was observed for the transition from double obesity-induced dysfunctions to all-cause death (fully adjusted HR: 0.86, 95% CI: 0.81–0.91). Among women, the strongest inverse association was noted for the transition from baseline to first obesity-induced dysfunction (fully adjusted HR: 0.81, 95% CI: 0.79–0.83).

The analysis based on grip strength tertiles further confirmed the observed associations. Table 5 presents the association between grip strength tertiles and preclinical obesity progression risk. A significant inverse trend was observed across all stages of preclinical obesity progression, with fully adjusted HRs decreasing significantly with increasing grip strength. Using the lowest tertile as the reference group, the highest grip strength levels consistently demonstrated a protective effect at each stage across all 3 trajectory models. Notably, this protective association was most prominent in model 1, with multivariable fully adjusted HRs for 3 transitions being 0.80 (95% CI: 0.79–0.82), 0.88 (95% CI: 0.85–0.90), and 0.77 (95% CI: 0.70–0.84), respectively.

**Figure 3.**
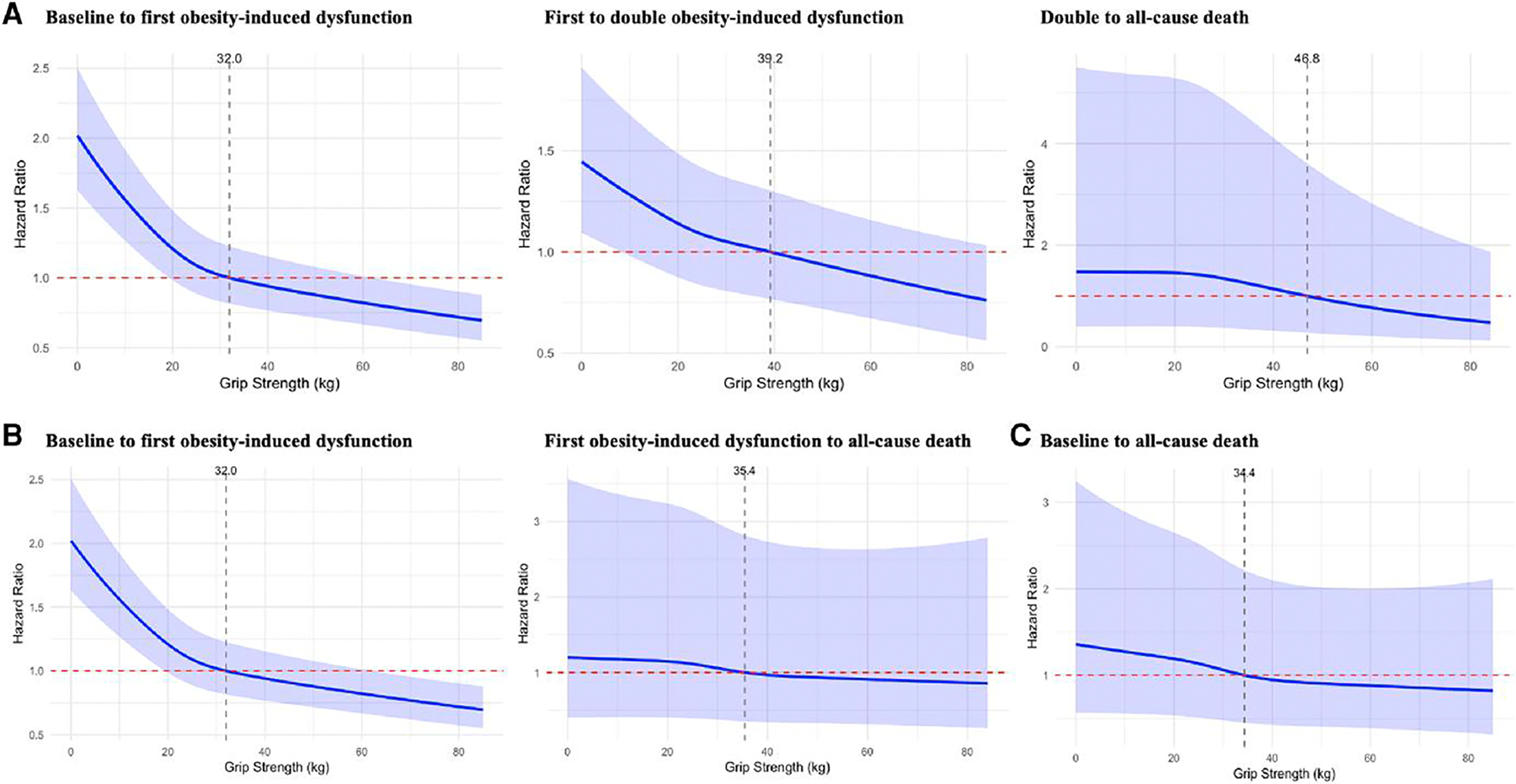
Nonlinear association between grip strength and obesity-induced dysfunction progression using restricted cubic splines. (A) Model 1: baseline to first obesity-induced dysfunction, first to double obesity-induced dysfunction, and double to all-cause death. (B) Model 2: baseline to first obesity-induced dysfunction, first obesity-induced dysfunction to all-cause death. (C) Model 3: baseline to all-cause death. All models were adjusted for age; sex; race; triglycerides, total cholesterol, low-density lipoprotein, high-density lipoprotein, hemoglobin A1c, systolic blood pressure, diastolic blood pressure, body mass index, waist circumference, estimated glomerular filtration rate, and C-reactive protein; family history of diabetes; education qualification; employment status; smoking status; alcohol drinking status; healthy diet; regular physical activity; sleep condition; vitamin supplements; and use of antihypertensive drugs, lipid-lowering drugs, and glucose-lowering drugs.

To further investigate the nonlinear association, we applied restricted cubic spline models to evaluate the potential nonlinear relationship between grip strength and the risks of obesity-induced dysfunctions progression and all-cause mortality (Fig. 3). Figure 3A to 3C depicts the results across the three multistate trajectory models. After comprehensive adjustment for potential confounders, the findings identified threshold values of grip strength associated with a reduction in risk across progressive stages of preclinical obesity. Specifically, in model 1 (Fig. 3A), the minimum grip strength was 32.0, 39.2, and 46.8 kg, respectively. In model 2 (Fig. 3B), protective thresholds were observed at 32.0 and 35.4 kg. In model 3 (Fig. 3C), the protective threshold was identified at 34.4 kg.

### Association of Grip Strength With CVD-specific and Cancer-specific Mortality

Table 6 presents the association between grip strength and the transition of preclinical obesity to cause-specific death. Each SD increase in grip strength was significantly associated with a lower risk of progressing from double obesity-induced dysfunction to CVD-specific and cancer-specific death (model 1), with fully adjusted HRs of 0.82 (95% CI: 0.77–0.88) and 0.91 (95% CI: 0.85–0.97), respectively. However, after multiple adjustments, grip strength was not significantly associated with the risk of transition from first obesity-induced dysfunction or from baseline to either cause-specific death among participants with preclinical obesity.

### Subgroup and Sensitivity Analyses

Stratified analyses of HRs for the association between grip strength and preclinical obesity progression risk in model 1 are presented in Table 7. Notably, the decreased dysfunction incidence and all-cause mortality risk associated with each SD increase in grip strength was more pronounced among individuals of Black race in the first progression (fully adjusted HR: 0.76, 95% CI: 0.63–0.92), women in the first progression (fully adjusted HR: 0.81, 95% CI: 0.79–0.83) and nonsmokers in the third progression (fully adjusted HR: 0.81, 95% CI: 0.74–0.87).

To ensure robustness, participants with complete data on either total thigh fat-free muscle volume measured by MRI (n = 6023) or total lean mass measured by DXA (n = 6380) were analyzed separately. The crucial role of MWR and LWR in the occurrence and progression of obesity-induced dysfunctions and all-cause death was demonstrated using a multistate model, as shown in Table 8. For each SD increase in MWR, the risk of disease progression at any stage in model 1 was significantly reduced. However, for each SD increase in LWR, a significant reduction in the risk of preclinical obesity was observed only in the first 2 progression stages of model 1 (fully adjusted HR: 0.94, 95% CI: 0.89–0.99 and fully adjusted HR: 0.87, 95% CI: 0.81–0.94, respectively).

## Discussion

In this large-scale prospective study, grip strength was significantly associated with the progression of obesity-induced dysfunctions and all-cause mortality. The inverse association was consistently observed in both men and women. Specifically, higher grip strength was associated with a significantly lower risk of transitioning from the stage of 2 or more obesity-induced dysfunctions to CVD-specific death and cancer-specific death. These associations remained robust in subgroup and sensitivity analyses, particularly during the transition from baseline to the first dysfunction.

At baseline, our study observed that with increasing tertiles of grip strength, WC, rather than BMI, showed a significant decreasing trend. Consistent with our findings, a previous study has demonstrated that muscle strength more accurately reflects body composition distribution, exhibiting a significant inverse association with abdominal obesity (23). Additionally, individuals with higher grip strength exhibited significantly decreased HbA1c and TG levels, suggesting a potential relationship between grip strength and improved glucose-lipid metabolic profiles. Similarly, a meta-analysis has shown that reduced grip strength is associated with an increased risk of type 2 diabetes, including elevated HbA1c levels (24). Our results also revealed that individuals in the highest grip strength tertile reported greater engagement in regular physical activity and a lower prevalence of past or current smoking. Importantly, smoking has been previously identified as a notable determinant of muscle strength decline (25). These findings suggest that muscle mass and function are positively associated with healthier lifestyle patterns.

Numerous studies have demonstrated a strong association between grip strength and a wide range of metabolic complications, as well as all-cause mortality (26, 27). Beyond investigating the effect of grip strength on the progression from preclinical to clinical obesity and mortality, our study also utilized a multistate model among participants with complete MRI and DXA data to assess the associations between total thigh fat-free muscle volume, total lean mass, and the risk of obesity-induced dysfunctions progression and all-cause mortality. Notably, our findings indicated that MWR exhibits a more pronounced inverse correlation with these risks compared to LWR, which encompasses additional soft tissue components. This finding might be attributed to a prior study suggesting that DXA tends to underestimate age-related muscle loss when compared to MRI (28).

The strong correlation observed between grip strength, MWR, and the progression of preclinical obesity and mortality may be explained by several plausible mechanisms. First, obesity-induced dysfunctions are primarily driven by excess adiposity, which directly impairs organs and tissues and contributes to limitations in ADL (9). Previous research has demonstrated a significant inverse association between grip strength and percentage body fat (29). The expansion of adipose tissue thus forms the biological basis for the development of dysfunctions. Second, our results revealed that individuals in the highest tertile of grip strength have significantly lower CRP levels. Similarly, previous evidence indicates that reduced muscle strength is significantly associated with elevated CRP levels (30). As a well-established biomarker of systemic inflammation, CRP has been linked to an increased risk of cardiovascular mortality and metabolic disorders, including metabolic-associated fatty liver disease (31, 32). Mechanistically, obesity is recognized as a chronic low-grade inflammatory state, wherein hypertrophied adipose tissue attracts immune cells, including monocytes and macrophages, which in turn promotes the secretion of proinflammatory cytokines such as tumor necrosis factor-α and interleukin-6, leading to local or systemic inflammation and oxidative stress (33, 34). These processes ultimately contribute to the progression of dysfunctions. Third, skeletal muscle itself is an active endocrine organ that secretes myokines such as irisin and interleukin-15, which play protective roles in metabolic homeostasis and insulin sensitivity (35, 36). Reduced muscle mass or strength may impair myokine secretion, leading to disrupted energy metabolism. Endocrine dysregulation is also a key mechanism contributing to obesity-induced dysfunctions (9). Additionally, reduced muscle strength may, in part, result from decreased bone mineral density (BMD). Previous research has suggested that reduced grip strength may reflect decreased BMD (37). Evidence suggested that normal bone remodeling and vascular calcification share common signaling pathways, and reduced BMD may contribute to CVD (38). Contributing factors to reduced BMD, such as vitamin D deficiency and hypogonadism, also have been suggested to contribute to the pathogenesis of chronic metabolic diseases (39). The role of BMD in the progression of obesity-induced dysfunctions and cause-related mortality still warrants further investigation in future studies.

The strength of this study lies in the application of a multistate model to comprehensively illustrate the role of grip strength across the trajectory of preclinical progression in different models. In addition, the UK Biobank not only provides a large sample size and a prolonged follow-up period but also ICD-10-coded diagnostic records that are traceable prior to baseline and continuously updated postenrollment, enabling accurate identification of baseline dysfunctions, subsequent disease transitions, and mortality outcomes. Nevertheless, several limitations should be acknowledged. First, as participants with missing data related to the definition of preclinical obesity and the main exposure (grip strength) were excluded, selection bias is inevitable in the present study. Second, although the 18 obesity-induced dysfunctions included in our definition were selected based on an expert consensus statement, these conditions have multifactorial origins and may not be exclusively attributable to obesity. Third, individuals with BMI < 30 but with excess central obesity indicators were not included in this study based on the definition criterion. Future studies are needed to validate these findings in broader populations. In addition, our analysis focused exclusively on individuals with baseline preclinical obesity, and due to the absence of longitudinal body composition data, we could not evaluate whether changes in obesity status influenced disease trajectories. Finally, we did not investigate the underlying biological mechanisms of grip strength across different models at the genomic or proteomic level, leaving the mechanistic association between grip strength and preclinical progression trajectories unresolved.

In conclusion, this study uniquely explored the relationship between grip strength and the longitudinal progression trajectories of preclinical obesity, characterized by elevated anthropometric parameters and evidence of excessive adiposity without established obesity-induced dysfunctions. Our study demonstrated for the first time that increased grip strength significantly mitigates the risk of progression from preclinical obesity to obesity-induced dysfunctions and subsequent mortality. Moreover, this protective association persisted across diverse anthropometric measures and sensitivity analyses utilizing alternative muscle-related metrics (MWR and LWR). These findings provide novel evidence emphasizing muscle strength improvement as a potential early intervention strategy for preventing adverse health outcomes associated with the preclinical obesity status.

## Figures and Tables

**Table 1.** List of dysfunctions due to obesity or limitations of daily activities by The Lancet Diabetes and Endocrinology Commission on definition and diagnostic criteria of clinical obesity (9)

Organ, tissue, or body system	Diagnostic criterion for dysfunctions due to obesity	ICD-10 codes

CNS	Signs of raised intracranial pressure such as vision loss and/or recurrent headaches	G93.2 and H54 or G93.2 and either R51, G43, and G44
Upper airways	Apnoeas/hypopnoeas during sleep due to increased upper airways resistance	G47
Respiratory	Hypoventilation and/or breathlessness and/or wheezing due to reduced lung and/or diaphragmatic compliance	J44 without previous smoking habit
Cardiovascular (ventricular)	Reduced left ventricular systolic function—heart failure with reduced ejection fraction	I50.2
Cardiovascular (atrial)	Chronic/recurrent atrial fibrillation	I48.2
Cardiovascular (pulmonary)	Pulmonary artery hypertension	I27
Cardiovascular	Chronic fatigue, lower limb edema due to impaired diastolic dysfunction–heart failure with preserved ejection fraction	I50.3
Cardiovascular (thrombosis)	Recurrent DVT and/or pulmonary thromboembolic disease	I26, I82
Cardiovascular (arterial)	Raised arterial blood pressure	I10
Metabolism	The cluster of hyperglycemia, high triglyceride levels, and low HDL cholesterol levels	R73, E11, E78
Liver	NAFLD with hepatic fibrosis	K75.81, K74.0, K76.0
Renal	Microalbuminuria with reduced eGFR	N18
Urinary	Recurrent/chronic urinary incontinence	N39.4, N39.3, R39
Reproductive (female)	Anovulation, oligomenorrhea, and PCOS	E28.2, N91, E28
Reproductive (male)	Male hypogonadism	E29
Musculoskeletal	Chronic, severe knee or hip pain associated with joint stiffness and reduced range of joint motion	M16, M17, M25.5, M25.65, M25.66
Lymphatic	Lower limbs lymphedema causing chronic pain and/or reduced range of motion	I89.0
Limitations of day-to-day activities	Significant, age-adjusted limitations of mobility and/or other basic activities of daily living (bathing, dressing, toileting, continence, eating)	R53.1, Z74, Z73.6

Abbreviations: CNS, central nervous system; DVT, deep vein thrombosis; eGFR, estimated glomerular filtration rate; HDL, high-density lipoprotein; ICD-10, International Classification of Diseases, 10th Revision; NAFLD, nonalcoholic fatty liver disease; PCOS, polycystic ovary syndrome.

**Table 2.** Baseline characteristics of the participants among grip strength tertiles from the UK Biobank

Variables	T1	T2	T3	*P*-values

No. of participants	31 571	31 630	30 074	
Age (years)	58.2 ± 7.50	56.3 ± 7.71	53.0 ± 7.73	<.001
Sex, n (%)				
Women	16 828 (53.3)	17 044 (53.9)	15 982 (53.1)	.147
Men	14 743 (46.7)	14 586 (46.1)	14 092 (46.9)	.147
Race, n (%)				
White	27 612 (87.5)	28 080 (88.8)	26 608 (88.5)	<.001
Black	205 (0.6)	131 (0.4)	103 (0.3)	<.001
Asian	1536 (4.9)	1701 (5.4)	1761 (5.9)	<.001
Other	2218 (7.0)	1718 (5.4)	1602 (5.3)	<.001
Family history of diabetes, n (%)	6473 (20.5)	6668 (21.1)	6586 (21.9)	<.001
Education, n (%)				
College or high degree	2732 (8.7)	2922 (9.2)	3157 (10.5)	<.001
Others	28 569 (90.5)	28 477 (90.0)	26 666 (88.7)	<.001
Employment, n (%)				
Currently employed	13 970 (44.2)	17 890 (56.6)	20 679 (68.8)	<.001
Others	17 537 (55.5)	13 717 (43.4)	9367 (31.1)	<.001
Body mass index (kg/m^2^)	33.7 ± 3.78	33.5 ± 3.65	33.6 ± 3.70	<.001
Waist circumference (cm)	105 ± 11.0	104±10.7	103 ± 10.6	<.001
Blood pressure (mmHg)				
Systolic	144 ± 19.2	144 ± 18.9	142 ± 18.6	<.001
Diastolic	85.4 ± 10.4	86.3 ± 10.2	86.9 ± 10.4	<.001
Sleep (hours/day)	7.04 ± 1.55	7.05 ± 1.34	7.03 ± 1.24	<.001
Hemoglobin A1c (mmol/mol)	38.4 ± 8.24	37.4 ± 7.49	36.5 ± 6.81	<.001
Total cholesterol (mmol/L)	5.65 ± 1.19	5.74 ± 1.16	5.76 ± 1.11	<.001
Low-density lipoprotein cholesterol (mmol/L)	3.56 ± 0.90	3.63 ± 0.87	3.67 ± 0.84	<.001
High-density lipoprotein cholesterol (mmol/L)	1.32 ± 0.34	1.33 ± 0.34	1.32 ± 0.33	<.001
Triglycerides (mmol/L)	2.11 ± 1.13	2.08 ± 1.14	2.07 ± 1.18	<.001
C-reactive protein (mg/L)^a^	2.63 (1.41–4.97)	2.40 (1.30–4.48)	2.15 (1.15–4.09)	<.001
Estimated glomerular filtration rate (mL/min/1.73 m^2^)	88.6 ± 13.0	89.3 ± 12.8	90.5 ± 12.8	<.001
Current or past smokers, n (%)	15 021 (47.6)	15 023 (47.5)	14 007 (46.6)	.022
Current drinkers, n (%)	27 663 (87.6)	29 003 (91.7)	27 915 (92.8)	<.001
Healthy diet, n (%)	12 574 (39.8)	12 437 (39.3)	11 404 (37.9)	<.001
Regular physical activity, n (%)	17 448 (55.3)	19 472 (61.6)	19 686 (65.5)	<.001
Vitamin supplement, n (%)	9553 (30.3)	9522 (30.1)	8816 (29.3)	.024
Use of medications, n (%)				
Lipid-lowering	4660 (14.8)	3622 (11.5)	2565 (8.5)	<.001
Antihypertensive	5081 (16.1)	4047 (12.8)	3018 (10.0)	<.001
Glucose-lowering	2254 (7.1)	1452 (4.6)	963 (3.2)	<.001

*P*-value: adjusted for age. T1, T2, and T3 represent sex-specific tertiles of grip strength: men: T1 (≤36.5 kg), T2 (36.5–44.0 kg), T3 (>44.0 kg); women: T1 (≤20.5 kg), T2 (20.5–26.0 kg), T3 (>26.0 kg).

a Data in median (interquartile range).

**Table 3.** Association between grip strength and preclinical obesity progression

	Simple adjusted HR (95% CI)	*P*-value	Multiple adjusted HR (95% CI)	*P*-value

Model 1				
Baseline to first dysfunction	0.81 (0.80–0.82)	<.001	0.86 (0.85–0.88)	<.001
First dysfunction to double dysfunctions	0.87 (0.85–0.88)	<.001	0.92 (0.90–0.94)	<.001
Double dysfunctions to all-cause death	0.82 (0.78–0.86)	<.001	0.87 (0.83–0.91)	<.001
Model 2				
Baseline to first dysfunction	0.81 (0.80–0.82)	<.001	0.86 (0.85–0.88)	<.001
First dysfunction to all-cause death	0.91 (0.86–0.98)	.007	0.93 (0.87–0.99)	.033
Model 3				
Baseline to all-cause death	0.87 (0.82–0.93)	<.001	0.91 (0.85–0.97)	.002

Grip strength was standardized as a *z*-score. One SD increase in grip strength corresponds to 11.60 kg.

Simple adjusted: adjusted for age, sex, and race.

Multiple adjusted: further adjusted for triglycerides, total cholesterol, low-density lipoprotein, high-density lipoprotein, hemoglobin A1c, systolic blood pressure, diastolic blood pressure, body mass index, waist circumference, estimated glomerular filtration rate, and C-reactive protein; family history of diabetes; education qualification; employment status; smoking status; alcohol drinking status; healthy diet; regular physical activity; sleep condition; vitamin supplement; and use of antihypertensive drugs, lipid-lowering drugs, and glucose-lowering drugs.

Abbreviations: CI, confidence interval; HR, hazard ratio.

**Table 4.** Association between grip strength and preclinical obesity progression by biological sex

	Simple adjusted HR (95% CI)	*P*-value	Multiple adjusted HR (95% CI)	*P*-value

Men				
Trajectory				
Baseline to first dysfunction	0.83 (0.83–0.85)	<.001	0.89 (0.88–0.91)	<.001
First dysfunction to double dysfunctions	0.88 (0.86–0.89)	<.001	0.93 (0.91–0.95)	<.001
Double dysfunctions to all-cause death	0.81 (0.77–0.85)	<.001	0.86 (0.81–0.91)	<.001
Women				
Trajectory				
Baseline to first dysfunction	0.76 (0.75–0.78)	<.001	0.81 (0.79–0.83)	<.001
First dysfunction to double dysfunctions	0.85 (0.82–0.88)	<.001	0.89 (0.86–0.92)	<.001
Double dysfunctions to all-cause death	0.85 (0.77–0.93)	<.001	0.90 (0.82–0.98)	.018

Grip strength was standardized as a z-score. One SD increase in grip strength corresponds to 11.60 kg.

Simple adjusted: adjusted for age, sex, and race.

Multiple adjusted: further adjusted for triglycerides, total cholesterol, low-density lipoprotein, high-density lipoprotein, hemoglobin A1c, systolic blood pressure, diastolic blood pressure, body mass index, waist circumference, estimated glomerular filtration rate, and C-reactive protein; family history of diabetes; education qualification; employment status; smoking status; alcohol drinking status; healthy diet; regular physical activity; sleep condition; vitamin supplement; and use of antihypertensive drugs, lipid-lowering drugs, and glucose-lowering drugs.

Abbreviations: CI, confidence interval; HR, hazard ratio.

**Table 5.** Association between grip strength tertiles and preclinical obesity progression

	T1 (n = 31 571)	T2 (n = 31 630)	*P*-value	T3 (n = 30 074)	*P*-value

Model 1					
Baseline to first dysfunction					
Number of events	19 400	16 931		13 885	
Multiple adjusted HR (95% CI)	1.00	0.88 (0.87–0.90)	<.001	0.80 (0.79–0.82)	<.001
First dysfunction to double dysfunctions					
Number of events	11 856	9328		6979	
Multiple adjusted HR (95% CI)	1.00	0.93 (0.91–0.96)	<.001	0.88 (0.85–0.90)	<.001
Double dysfunctions to all-cause death					
Number of events	2005	1255		686	
Multiple adjusted HR (95% CI)	1.00	0.90 (0.84–0.97)	.003	0.77 (0.70–0.84)	<.001
Model 2					
Baseline to first dysfunction					
Number of events	19 400	16 931		13 885	
Multiple adjusted HR (95% CI)	1.00	0.88 (0.87–0.90)	<.001	0.80 (0.79–0.82)	<.001
First dysfunction to all-cause death					
Number of events	898	691		463	
Multiple adjusted HR (95% CI)	1.00	0.95 (0.86–1.05)	.286	0.87 (0.78–0.98)	.021
Model 3					
Baseline to all-cause death					
Number of events	856	736		573	
Multiple adjusted HR (95% CI)	1.00	0.94 (0.85–1.03)	.192	0.87 (0.78–0.97)	.014

T1, T2, and T3 represent sex-specific tertiles of grip strength: men: T1 (≤36.5 kg), T2 (36.5–44.0 kg), T3 (>44.0 kg); women: T1 (≤20.5 kg), T2 (20.5–26.0 kg), T3 (>26.0 kg).

Multiple adjusted: further adjusted for triglycerides, total cholesterol, low-density lipoprotein, high-density lipoprotein, hemoglobin A1c, systolic blood pressure, diastolic blood pressure, body mass index, waist circumference, estimated glomerular filtration rate, and C-reactive protein; family history of diabetes; education qualification; employment status; smoking status; alcohol drinking status; healthy diet; regular physical activity; sleep condition; vitamin supplement; and use of antihypertensive drugs, lipid-lowering drugs, and glucose-lowering drugs.

Abbreviations: CI, confidence interval; HR, hazard ratio.

**Table 6.** The role of grip strength in transition of obesity-induced dysfunction to specific-cause death

	Simple adjusted HR (95% CI)	*P*-value	Multiple adjusted HR (95% CI)	*P*-value

CVD-specific death				
Model 1				
Double dysfunctions to cause-specific death	0.76 (0.71–0.81)	<.001	0.82 (0.77–0.88)	<.001
Model 2				
First dysfunction to cause-specific death	0.90 (0.81–1.01)	.064	0.93 (0.83–1.05)	.234
Model 3				
Baseline to cause-specific death	0.89 (0.81–0.99)	.030	0.96 (0.86–1.06)	.425
Cancer-specific death				
Model 1				
Double dysfunctions to cause-specific death	0.89 (0.83–0.95)	<.001	0.91 (0.85–0.97)	.004
Model 2				
First dysfunction to cause-specific death	1.01 (1.03–1.10)	.862	1.03 (0.95–1.12)	.482
Model 3				
Baseline to cause-specific death	0.93 (0.85–1.02)	.123	0.94 (0.86–1.02)	.151

Grip strength was standardized as a *z*-score. One SD increase in grip strength corresponds to 11.60 kg.

Model 1: Double obesity-induced dysfunction to specific-cause death.

Model 2: First obesity-induced dysfunction to specific-cause death.

Model 3: Baseline to cause-specific death.

Simple adjusted: adjusted for age, sex, and race.

Multiple adjusted: **f**urther adjusted for triglycerides, total cholesterol, low-density lipoprotein, high-density lipoprotein, hemoglobin Alc, systolic blood pressure, diastolic blood pressure, body mass index, waist circumference, estimated glomerular filtration rate, and C-reactive protein; family history of diabetes; education qualification; employment status; smoking status; alcohol drinking status; healthy diet; regular physical activity; sleep condition; vitamin supplement; and use of antihypertensive drugs, lipid-lowering drugs, and glucose-lowering drugs.

Abbreviations: CI, confidence interval; CVD, cardiovascular disease; HR, hazard ratio.

**Table 7.** Subgroup analysis of the association between grip strength and preclinical obesity progression

Variables	Baseline to first dysfunction	First dysfunction to double dysfunctions	Double dysfunctions to death

Sex			
Women	0.81 (0.79–0.83)	0.89 (0.86–0.92)	0.90 (0.82–0.98)
Men	0.86 (0.85–0.88)	0.89 (0.87–0.91)	0.84 (0.80–0.89)
Age			
<45 years	0.86 (0.82–0.91)	0.91 (0.85–0.98)	0.79 (0.56–1.12)
45–65 years	0.84 (0.83–0.86)	0.88 (0.87–0.90)	0.85 (0.80–0.90)
≥65 years	0.85 (0.83–0.88)	0.91 (0.88–0.95)	0.88 (0.82–0.95)
Race			
White	0.84 (0.83–0.85)	0.89 (0.87–0.91)	0.85 (0.81–0.90)
Black	0.76 (0.63–0.92)	0.80 (0.64–1.00)	0.69 (0.30–1.60)
Asian	0.83 (0.78–0.89)	0.94 (0.86–1.03)	0.87 (0.65–1.15)
Others	0.89 (0.84–0.93)	0.90 (0.84–0.96)	0.93 (0.76–1.13)
Education qualification			
Others	0.84 (0.83–0.86)	0.89 (0.87–0.91)	0.86 (0.82–0.90)
College or high degree	0.85 (0.81–0.89)	0.91 (0.86–0.97)	0.83 (0.70–0.98)
Employment status			
Others	0.84 (0.82–0.85)	0.90 (0.88–0.92)	0.87 (0.83–0.92)
Currently employed	0.85 (0.84–0.87)	0.88 (0.86–0.90)	0.83 (0.76–0.90)
Smoking			
Other	0.83 (0.81–0.85)	0.87 (0.85–0.89)	0.81 (0.74–0.87)
Current or past smoker	0.86 (0.84–0.87)	0.91 (0.89–0.93)	0.88 (0.84–0.94)
Physical activity			
<150 minutes/week	0.84 (0.82–0.85)	0.90 (0.87–0.92)	0.88 (0.83–0.94)
≥150 minutes/week	0.85 (0.83–0.86)	0.89 (0.87–0.91)	0.83 (0.78–0.89)
Drinking			
Other	0.82 (0.79–0.85)	0.89 (0.85–0.94)	0.87 (0.76–0.99)
Current drinker	0.85 (0.83–0.86)	0.89 (0.87–0.91)	0.86 (0.81–0.90)
Healthy diet			
No	0.84 (0.83–0.86)	0.89 (0.87–0.91)	0.84 (0.79–0.90)
Yes	0.84 (0.83–0.86)	0.89 (0.87–0.92)	0.88 (0.81–0.94)
Vitamins supplement			
No	0.84 (0.83–0.86)	0.90 (0.88–0.91)	0.83 (0.78–0.88)
Yes	0.85 (0.83–0.87)	0.88 (0.85–0.91)	0.93 (0.85–1.01)
Family history of diabetes			
No	0.85 (0.84–0.86)	0.89 (0.87–0.91)	0.86 (0.82–0.91)
Yes	0.83 (0.80–0.85)	0.90 (0.87–0.94)	0.84 (0.76–0.93)
Sleep duration			
< 6	0.85 (0.82–0.89)	0.92 (0.86–0.97)	0.97 (0.84–1.13)
≥ 6	0.84 (0.83–0.86)	0.89 (0.87–0.91)	0.85 (0.81–0.89)

**Table 8.** Sensitivity analysis of the association between the MWR or LWR and preclinical obesity progression

	Simple adjusted HR (95% CI)	*P*-values	Multiple adjusted HR (95% CI)	*P*-values

Model 1				
MWR				
Baseline to first dysfunction	0.76 (0.72–0.80)	<.001	0.83 (0.78–0.88)	<.001
First dysfunction to double dysfunctions	0.82 (0.76–0.89)	<.001	0.88 (0.81–0.96)	.003
Double dysfunctions to all-cause death	0.64 (0.45–0.91)	.012	0.64 (0.44–0.95)	.025
LWR				
Baseline to first dysfunction	0.88 (0.84–0.93)	<.001	0.94 (0.89–0.99)	.036
First dysfunction to double dysfunctions	0.83 (0.76–0.89)	<.001	0.87 (0.81–0.94)	.001
Double dysfunctions to all-cause death	0.93 (0.66–1.31)	.678	0.96 (0.67–1.37)	.828
Model 2				
MWR				
Baseline to first dysfunction	0.76 (0.72–0.80)	<.001	0.83 (0.78–0.88)	<.001
First dysfunction to all-cause death	0.88 (0.57–1.36)	.555	0.84 (0.54–1.33)	.462
LWR				
Baseline to first dysfunction	0.88 (0.84–0.93)	<.001	0.95 (0.90–1.00)	.062
First dysfunction to all-cause death	0.84 (0.49–1.42)	.507	0.85 (0.49–1.47)	.556
Model 3				
MWR				
Baseline to all-cause death	1.14 (0.70–1.85)	.606	1.21 (0.74–1.97)	.450
LWR				
Baseline to all-cause death	1.26 (0.76–2.10)	.364	1.28 (0.76–2.14)	.349

MWR was standardized as a *z*-score. One SD increase in MWR corresponds to 0.02.

LWR was standardized as a *z*-score. One SD increase in LWR corresponds to 0.07.

Simple adjusted: adjusted for age, sex, and race.

Multiple adjusted: further adjusted for family history of diabetes; education qualification; employment status; smoking status; alcohol drinking status; healthy diet; regular physical activity; sleep condition; vitamin supplement; and use of antihypertensive drugs, lipid-lowering drugs, and glucose-lowering drugs.

Participants in analysis of the association between the MWR and the trajectory of obesity-induced dysfunction multimorbidity: 6023.

Participants in analysis of the association between the LWR and the trajectory of obesity-induced dysfunction multimorbidity: 6380.

Abbreviations: LWR, lean mass-weight ratio; MWR, muscle-weight ratio.

## Data Availability

The raw UK Biobank data are protected and are not available due to data privacy laws. Researchers can apply to use the UK Biobank resource for health-related research and public interest via the UK Biobank Access Management System (https://ams.ukbiobank.ac.uk/ams/).
